# Perspectives of Hospitalized Mental Health Care Users Concerning the Involvement of Family Members in Their Care: A Qualitative Study

**DOI:** 10.3390/nursrep13040139

**Published:** 2023-12-04

**Authors:** Mihloti E. Shimange, Hilda N. Shilubane

**Affiliations:** Department of Advanced Nursing Science, Faculty of Health Sciences, University of Venda, Thohoyandou 0950, South Africa; shim3b@gmail.com

**Keywords:** care, family members, involvement, hospitalized, mental health care users, perspectives

## Abstract

The value of families and professionals in mental health care is well understood. Patient perspectives appear to have gotten less attention to date. This study investigated the perspectives of hospitalized mental health care users on the involvement of family members in their care using a qualitative phenomenological design. The participants with lived experience of family members being involved in their care were chosen using non-probability, purposive sampling. Individual interviews were carried out with the assistance of a voice recorder and observation notes. Because of data saturation, only fifteen people were interviewed. The interviews were transcribed verbatim and analyzed using Colaizzi’s method. It started with reading and reviewing the transcript to extract key statements about the phenomenon. The meaning was then determined by carefully studying the primary significant statements and phrases. The established meanings were then organized into themes and subthemes. The three themes that emerged from the data analysis are as follows: a wide variation in patients’ perspectives when family members remind them of their medicine, unpredictable visitation by family members, and a lack of support from family members. There were also the following five sub-themes: not visiting mental health care users in the hospital causes uncertainty about their future, mental health care users were concerned by fewer visits from relatives, friends were perceived as a contributory factor to no or limited visitation by family members, disappointment by a lack of financial support, and perceived lack of emotional support from family members. There is a need for family members to visit mental health care users to remove uncertainty about their future. Healthcare providers should listen to mental health care users to identify the challenges they are faced with, and hospital policies tailored to enhancing the involvement of family members should be formulated.

## 1. Introduction

Globally, there is a growing trend toward involving family members in patient care to smooth the transition from acute care to aftercare [1], and evidence suggests that incorporating relatives in the treatment plan of the patient enhances patient compliance with treatment [1,2]. Family involvement refers to family members’ explicit participation in the care planning of mental health care users (MHCUs). Research demonstrates that family engagement is a vital element in the management and rehabilitation of individuals with psychological disorders, as psychological issues are linked with a range of societal and emotional practices within the family. It has been noted that family participation helps mental health care users prevent relapses and spend less time in hospitals [3].

Scientific evidence, as well as legal and ethical considerations, supports family involvement for people with mental illnesses, and it is recommended in clinical practice guidelines. Family members play important roles in the care of patients including contributing to decision-making, assisting in home care, and addressing the expectations of patients, families, and society at large [4]. Although family members do assist in the care of MHCUs, it is disturbing to find that users do not receive a penny from their social grants. In South Africa, for example, the majority of MHCUs receive mental disability grants, and many households rely totally on this due to poverty caused by a lack of employment. This could aggravate MHCUs’ poor mental health while also delaying their rehabilitation. Despite the call by different international bodies to increase family involvement, there is no agreement as to what this involvement means and how it should take place [5].

A major barrier to effective family involvement in health care has been identified as a lack of adequate information and cooperation from mental health services [6]. Mental health specialists in South Africa have constantly said that the major obstacles are a lack of implementation plans, staff shortages, and inadequate mental health funds [7].

Engaging family members can lead to decreased setbacks, decreased admissions, reduced hospital stays, and better compliance with treatment. Relatives play a significant part in assisting MHCUs and encouraging improved coping skills [3,8]. Family involvement aids in the early recognition of symptoms of relapse in mental health care users diagnosed with schizophrenia [9]. Relatives’ perceptions regarding the care of patients are crucial according to the study by Mabunda [3], as reduced interactions with MHCUs by family members can lead to health problems and increased hospitalization rates. Regardless of the potential advantages of involving members of the family in the treatment of healthcare users, numerous reports have emerged highlighting the challenges of executing family participation in everyday care.

Similarly, another study recommends improving mental health services by considering the perspectives of consumers and caregivers [10], and this is supported by the government and mental health professionals [11]. Allowing users to have views regarding their care somehow gives them peace of mind and understanding because they have a platform to verbalize their concerns. Mental health care users emphasize the significance of mental health professionals discussing diagnoses with patients and caregivers, as well as providing hope and information about their diagnosis [12]. In terms of treatment planning, users want to be involved in their treatment decisions as well as their caregivers, but they are frequently excluded from decision-making [13]. Furthermore, users report being unprepared for hospital discharge, not being involved in the decision, and not receiving adequate post-discharge support together with their caregivers [14].

MHCUs require a substantial amount of help from family members [15]. The presence of family members and their involvement in caring for acutely ill adult inpatients is a widespread practice in African countries [16]. Similarly, due to cultural expectations [17,18], family members provide any care that the nurses miss, guaranteeing that their patients’ requirements are addressed. The National Mental Health Policy Framework and Strategic Plan also stresses the right to practice culturally acceptable religious practices and spiritual beliefs [19].

One effective technique for encouraging family members’ participation, according to a study published by Lloyd and King [6], is to perform periodic evaluations on inpatient treatment to highlight areas where services might be improved. However, MHCUs’ opinions of the role of family members in their care are uncertain. Positive family participation necessitates the participation of both the MHCUs’ family members and healthcare professionals [20]. There are no policies, regulations, or guidelines on the involvement of family members in the care of MHCUs. As a beginning point, the patient’s preference for family involvement is suggested [21]. The value of families and professionals in mental health care is well understood. A consensus from patient perspectives is now required, as this area appears to have received less attention to date [22]. This study attempts to fill that gap by investigating hospitalized MHCUs’ perspectives on the participation of family members in their care.

## 2. Methods

### 2.1. Study Design

A qualitative phenomenological research design was adopted in this study. The researcher chose this design because it allows the researcher to gain a thorough knowledge of the phenomenon being investigated, which is the opinions of MHCUs regarding the family member’s participation in their treatment plan [23].

### 2.2. Participants and Sampling

Eligibility for participation included the age of 18 years and above with a diagnosis of mental illness, being stable, able to communicate clearly, and being admitted to the three specialized mental health establishments or psychiatric hospitals in Limpopo province. Purposive sampling was used to select MHCUs whose family members accompanied them for healthcare services, visited them while admitted, supervised them on taking of oral treatment following their discharge, and provided any form of support to the user. Twenty-one mental health care users who met the inclusion criteria were approached for participation and all agreed; fifteen of them were males and six were females. Participants were recruited through the operational managers of the units on behalf of the researcher.

### 2.3. Data Collection

The researcher made an appointment with the prospective participants in their wards and requested permission from the nursing staff to conduct the interviews. Participants were invited to the private consultation room where interviews were to take place. A detailed explanation of the essence and reason for the research was provided to the members and permission was sought in writing before the commencement of the interviews. Although twenty-one participants were sampled, individual interviews were conducted with 15 MHCUs only due to data saturation. Data were collected from July to September 2022. The interviews were conducted in the designated health establishments in Limpopo province by the first author and the participant alone in the interview room for 35 to 50 min. There was no participant withdrawal, and the researcher did not have a prior relationship with the participants. The discussions were taped with a recording device, and notes were kept at all times. The interviews began by eliciting the participants to introduce themselves. This was followed by brief demographic information to provide context for the data collected. The first author, a female professional nurse, and a Ph.D. candidate asked the following central question to the participants: What is your perspective regarding the participation of your relatives in your care? This was followed by follow-up questions based on the participants’ responses. The question was interpreted into three different dialects which were Tshivenda, Xitsonga, and Sesotho, which are the languages spoken in the area. The researcher made certain that all three languages were used so that the participants could feel at ease while taking part in the study. When the transcripts were made available to the participants, none of them took advantage of them.

### 2.4. Data Analysis

The first author transcribed and translated the audio-recorded data into English before beginning the analysis. Data were cross-checked and compared during this process to ensure the legitimacy of the transcripts. The second author then double-checked the correctness and gist of the transcripts. Colaizzi’s seven steps of data analysis were used [24]. It began with reading and rereading the transcript. The second step was to extract significant statements related to the phenomenon. Then, by carefully considering the main relevant statements and phrases, the meaning was arrived at. Following that, formulated meanings were grouped into themes and subthemes. Later, an in-depth description of the structure of the phenomenon was developed. The fundamental structure of the phenomenon was then described. The last step was to validate the findings using feedback from participants on the analyses [25]. At first, the data revealed six themes and four sub-themes. The themes were revisited by the first (MES) and second (NHS) authors which resulted in the three themes and five subthemes discussed below. Lastly, the themes were reviewed by both authors, and an independent researcher confirmed the findings, and the sentence structure was not altered to preserve exact quotes.

### 2.5. Trustworthiness

Guba’s model was used to maintain trustworthiness throughout this study [26,27]. The discussions were expanded as needed to ensure reliability and to allow the first author to devote extra time together with the participants, building relationships and confidence. Triangulation was used, which involved conducting individual interviews while writing down some observation notes. Investigator triangulation was conducted by involving both authors and the co-coder to analyze the data. A thorough explanation of the research methods, as well as the literature, was used to assess generalizability to other settings.

### 2.6. Ethical Consideration

The first author received ethical clearance from the ethics committee of the institution (FHS/21/PDC/16/1207), the Limpopo province gave permission (lp 2022-05-008), and the participating hospitals. Informed consent, confidentiality, privacy, voluntary participation, and autonomy were all upheld as ethical principles. Before the interview, all participants were notified of the purpose, and that their contribution was non-mandatory, with no repercussions should they opt to quit participating in the discussion. Participants were told that their experiences and perspectives would be kept private, and transcriptions anonymized (P1, P2, etc.) to aid in de-identification. Participants were also informed of the duration of the interviews. On the day of the interview, each interviewee was given an identifying number and asked to sign and return a written consent form.

## 3. Results

### 3.1. Demographic Characteristics of Participants

Table 1 indicates the demographic characteristics of the participants. There were nine males and six females, and their ages ranged from 21 to 70 years. See the table for more information.

### 3.2. Themes That Emerged from Data Analysis

Three themes and five subthemes emerged from the analysis of data collected from mental health care users, see Table 2 below.

#### 3.2.1. Theme 1: Wide Variation in Patients’ Perspectives When Family Members Remind Them of Their Treatment

Participants stated that their family members do care about them since they constantly reminded them of their therapy and expressed concern about their whereabouts. They went on to say that their parents inquiring about their treatment indicates how much they care about them and do not want them to relapse. The following quote attests to this.

“My parents do make follow-ups about my treatment. For example, if you find that the sun sets while I am not at home, they will call to find out my whereabouts because they know that I have not eaten anything nor taken my evening treatment. They call to remind me about treatment because they do not want me to relapse. They know that once I relapse, I beat people without sound reason, and it creates problems for them?”

(P15)

Another patient showed how a family member interrupted his conversation with the doctor in the doctor’s consultation room.

“When we enter the doctor’s consultation room, he asks me about my treatment. While busy talking with the doctor, she gets involved in the conversation. So, as I was explaining my version, she was explaining something I do not know, and the doctor wrote what she said instead of what I told him”.

(P5)

Some participants showed appreciation for the family members’ participation in their care. They further revealed their unhappiness when family members did not frequently visit them while they were in the hospital.

“My family participates in my care; the only problem is that they rarely visit. If I have something that bothers me, the time they decide to come and see me, I talk to them. The problem is that I cannot stay with them because I do not have anyone to look after me when they go to work”.

(P4)

#### 3.2.2. Theme 2: Unpredictable Visitation by Family Members

Participants had different perspectives on the nature of hospital visits by family members. Some reported having few visitors, while others reported not receiving visitors at all.

##### 3.2.2.1. Sub-Theme 2.1: Not Visiting MHCUs in the Hospital Causes Uncertainty about Their Future

The participants reported a lack of visitation by family members while admitted to the health establishments. Participants further mentioned that the behavior of not having visitors affects them. Participants had the following to say:

“Since I was admitted in this hospital my family members have never visited me nor come to the hospital to talk to staff members. But the time I was admitted to an acute care hospital they came once”.

(P2)

Another participant revealed uncertainty about her future if she happens to be discharged. She was worried because she did not know where to go after her discharge.

“Now that I’m in the hospital no one is coming and I don’t feel good because as of now, I don’t even know what will happen if I get discharged, since no one is visiting or supporting me”.

(P13)

Some participants were longing to see their children since it was school holidays, and these children were not visiting their parents. One participant was concerned when the child was not visiting him during the school holidays. This attests to the following,

“I have not seen my child for some time. I know that grade 12 learners have hectic days because they attend classes even on weekends”, (P9), and he further said, “But now they’re closed for the holidays, and I do not know what the problem could be for not coming to see me”.

Another participant blamed his behavior of abusing substances as the cause of the family members’ attitude towards him.

“My uncle disowned me because I smoke a lot of marijuana. The reason I came here this time is that after I was discharged from Hospital B, they called my uncle but when he came to the hospital, he told the nurses that he no longer wanted to stay with me hence they transferred me to this hospital”.

(P3)

##### 3.2.2.2. Sub-Theme 2.2: MHCU’s Concern with Unexplained Fewer Visits by Relatives

Some participants reported that family members came once to see them since they were admitted to the institution and then never returned. They were concerned when family members were not visiting them, particularly when they did not know the reason that kept them away.

“Uhm, my younger sister came with her husband to see me here at the hospital and it was my first time to meet her husband. And that was the only time I saw her coming here at the hospital to see me with that man and they never came back again”.

(P10)

##### 3.2.2.3. Sub-Theme 2.3: Friends Were Perceived as a Contributory Factor to No or Limited Visitation of MHCUs by Family Members

During the interviews, participants mentioned that their peers influenced them to do wrong things. They did not smoke or drink but interacting with bad friends led them to be involved with substances. Some acknowledged that their behavior was disturbing or frustrating to the family and could be the cause of family members not visiting them while in the hospital.

“Now I told myself that I should stay away from friends who are not in my class because when I grew up, I was not a person who smoked, or drank alcohol, I used to stay at home or go play soccer. Currently, I drink because I socialize with people who are always going to taverns and drinking alcohol, which could be the cause that makes my family not visit me”.

(P11)

Other participants mentioned that friends introduced them to substances, and some ended up defaulting on their treatment. Below is what the participants have to say:

“Sometimes when I am sitting at home and my old friends pass by our house. They will ask me why I am quiet and that is when I will be convinced to go out with them to have fun. Sometimes they buy me food or something that I need, then later they will say let us go and smoke marijuana and then I relapse, that is why I say I do not know what is wrong with me”.

(P6)

“We were smoking as a group and we were many, so when it was finished for me, the other one will take out hers and everyone took out just like that, we share all of us”.

(P4)

#### 3.2.3. Theme 3: Lack of Support from Family Members

##### 3.2.3.1. Sub-Theme 3.1 Disappointment by Lack of Financial Support

Participants mentioned that they do not receive financial support from family members. The following excerpt clearly shows that the participant was disappointed when his sibling could not give him the little money he requested, although he is financially stable:

“Eish with my brother it is hard for him to support me because I do not understand what is going on with him because he is working but he cannot give me a mere R10 even if I ask him for it”.

(P1)

##### 3.2.3.2. Sub-Theme 3.2 Perceived Lack of Emotional Support from Family Members

It is clear from the quotes that some participants were not emotionally supported by their family members. Not receiving visitors could mean you do not have a shoulder to lean on, and this made them not feel loved and supported.

“I am tired of being hospitalized because I do not have friends to interact with, and my family members do not visit me either. Even my boyfriend who introduced me to drugs does not visit me. I wish the doctor could discharge me so I can meet my friends at home”.

(P10)

“Uhm, ok… I mean my uncles do not come to see me, and they do not want me to go home. One of them works in Gauteng and he comes back every month end, meaning that if he were serious about me, he would be coming here to see me whenever he comes back from Johannesburg and even apply for my discharge”.

(P13)

“Oh, I do not feel good because as of now, I do not even know if I will get discharged or not, and no one is visiting or supporting me. The only time I saw them was during my admission, they said they would come and fetch me, but they did not come till today. I have not seen them for days”.

(P7)

## 4. Discussion

Our results describe mental health care users’ perspectives in three themes and five subthemes. Recently, family members have appeared as significant supporters of the team responsible for the well-being of the patient. In addition to assisting with daily tasks, they often accomplish a variety of other tasks related to the patient’s daily life [28]. As the shift from hospital to community care is challenging with additional risks to MHCUs’ mental health and psychological well-being, relatives should assist their hospitalized family members.

The current findings revealed that participants reported that some family members are involved in the care of MHCUs, and this act is appreciated by participants. For MHCUs, the inclusion of relatives in their management is seen as essential and is cherished. In mental health establishments, health professionals depend on family members for information about the progress of the discharged MHCU’s condition. Non-participation of relatives in the treatment of MHCUs could have a detrimental effect on the user as healthcare providers might not be informed of the condition of the user following the discharge, whether the treatment is effective or not.

Our results also show that some of the family members do not visit MHCUs while in the hospital and this causes uncertainty about their future. Several reasons could lead to family members not visiting the MHCUs. One participant indicated his behavior of abusing substances as a cause of his rejection by family members. Indeed, family members are reluctant to stay with MHCUs who abuse substances because it is challenging to care for such patients. This is in line with findings of studies that found substance use among people experiencing mental illnesses to be linked to inconsiderate and behavioral challenges [29,30]. It is not surprising to hear that some MHCUs in this study were abandoned in health establishments by family members. This could be due to their undesirable behaviors that made family members want to cut the bond they had with them. No visitation by family members could suggest that users do not have social and emotional support from family members, whereas Nie and colleagues [31] and Singo and Shilubane [32] perceived family support as more important in alleviating stress. As the cause of users’ mental illness is not known, support through visitation by family members is vital as they struggle to cope with their diagnoses and concerns about not being discharged from the hospital.

The current findings revealed that MHCUs were socializing with bad friends and this interfered with treatment adherence and contributed to their relapse. A study by King et al. [33] found close friendships to be associated with increased self-esteem and psychosocial adjustment. Most theoretical and qualitative studies propose mechanisms for friendship’s numerous benefits. Friendship often adds comfort, intimacy, and nourishment. In contrast, friends in the current study contributed to deviant behavior such as substance abuse which led to nonadherence to treatment and relapse [15].

Similar to our findings, some authors found that participants were not listened to by both healthcare professionals and family members. Often, they were interrupted in their conversation with healthcare providers [34,35]. This could have a detrimental effect on the user’s health and could even lead to death as important concerns about treatment such as drug side effects might not be addressed. Doherr et al. [36] demonstrated that shared decision-making might lead to a mutual agreement on treatment and long-term decisions, therefore, ignoring the concerns of the patient might hinder this.

Furthermore, participants expressed disappointment when family members who had income were unable to provide financial assistance. Such a concern may exacerbate their poor mental well-being, as Wollburg et al. [37] proved that providing money to healthcare users can help alleviate mental problems such as depression and anxiety disorders. Not giving even nominal financial support to MHCUs could cause self-esteem loss and negative activities such as crime. It should be noted that in African culture, support is crucial, be it emotional, financial, or physical, and a lack of support from family members could lead to poor adherence to treatment and service utilization after users’ discharge from the health institution [38,39].

## 5. Strengths and Limitations

The use of in-depth interviews allowed MHCUs to explore their perspectives on family involvement with little interviewer influence.

One potential limitation of this study is the small sample size; therefore, we cannot generalize the findings to all MHCUs. Since this study was conducted on hospitalized MHCUs, discharged MHCUs may be recruited and interviewed to supplement the findings of this research. Participants from various settings may have different perspectives on family involvement. Interviewing patients’ therapists and healthcare providers could have provided information to better contextualize the patients’ experiences.

## 6. Conclusions

This research increased our awareness of MHCUs’ perspective on family members’ participation in their care. This study calls for healthcare professionals to listen to MHCUs to identify challenges that could lead to treatment non-adherence and relapse. The study suggests that MHCUs should be given a little money from their social grants to buy things that they need and be emotionally supported by being visited while admitted; this might assist in the recovery of their mental disorders. To increase the opportunity for visitation by family members, healthcare providers should be focused and receptive to culturally diverse families, and services should be based on the preferences of MHCUs and their families. In addition, mental health outcomes in African MHCUs should be improved by reframing culturally competent care. Future studies will benefit from examining different provinces using a longitudinal design. This approach can clarify the differences between provinces to promote the formulation of hospital policies tailored to enhancing the involvement of family members in the care of South African MHCUs.

## Figures and Tables

**Table 1:** Participants’ demographic information (n = 15).

	Gender	Age	Grade Passed	Diagnosis	Duration of Illness	No. of Hospitalization
1	Male	42	10	Schizophrenia	23 Years	2
2	Male	26	9	Schizophrenia	7 Years	2
3	Male	62	7	SIPD	20 Years	2
4	Female	21	9	SIPD	3 years	3
5	Male	66	8	Schizophrenia	38 Years	2
6	Male	42	11	BMD	13 years	2
7	Male	70	2	Schizophrenia	Unsure	2
8	Female	46	6	Schizophrenia	31 Years	2
9	Male	62	9	BMD	23 Years	2
10	Female	34	3	Schizophrenia	9 Years	2
11	Male	35	10	SIPD	15 Years	2
12	Female	56	8	Schizophrenia	23 Years	2
13	Female	30	11	Schizophrenia	6 Years	2
14	Female	28	12	SIPD	5 Years	2
15	Male	24	11	Schizophrenia	9 Years	2

Note: SIPD = Substance-induced psychotic disorder, BMD = Bipolar mood disorder.

**Table 2:** Themes and subthemes.

Wide variation in patients’ perspectives when family members remind them of their treatment.
2. Unpredictable visitation by family members
a. Not visiting MHCUs in the hospital causes uncertainty about their future.
b. MHCU’s concern with unexplained fewer visits by relatives
c. Friends were perceived as a contributory factor to no or limited visitation of MHCUs by family members.
3. Lack of support from family members
a. Disappointment by lack of financial support
b. Perceived lack of emotional support from family members

## Data Availability

Data are available on request from the first author.

## Public Involvement Statement

No public involvement in any aspect of this research.

## Guidelines and Standards Statement

Throughout the study, we followed the COREQ Standards for Reporting Qualitative Research Guidelines.
